# CelluBase: a comprehensive genomic platform for advancing cellulose-producing bacteria

**DOI:** 10.1099/mgen.0.001825

**Published:** 2026-09-15

**Authors:** Bashir A. Akhoon, Lakshay Anand, Kendall R. Corbin

**Affiliations:** 1 Department of Horticulture, Martin-Gatton College of Agriculture, Food and Environment, University of Kentucky, Lexington, Kentucky, USA

**Keywords:** bacterial cellulose biosynthesis, biotechnology, CelluBase, cellulose synthase genes, database, synthetic biology

## Abstract

Bacterial cellulose (BC)-producing taxa constitute a functionally coherent yet phylogenetically heterogeneous assemblage of acetic acid bacteria distributed across evolutionary lineages within Alphaproteobacteria. Despite the importance of BC as a biomaterial in diverse biotechnological applications, a dedicated resource for the study and comparative analysis of cellulose-producing bacteria is currently lacking. To address this gap, we developed CelluBase – an integrated, user-friendly database for the study of bacteria from the genera *Komagataeibacter* and *Novacetimonas*. CelluBase is a domain-specific database dedicated to the genetic and functional dissection of BC synthesis. It integrates high-quality genome assemblies, functional annotations, operonic architectural analyses, multi-omics datasets and comparative genomics analyses for investigating the molecular mechanisms governing BC biosynthesis. Key features of the database include characterization of cellulose synthase gene organization, regulatory network architecture, metabolic pathway diversification and phylogenomic relationships across cellulose-producing lineages. Results generated within the CelluBase framework are available as text, interactive plots, and publication-ready figures. CelluBase (https://cellubase.shinyapps.io/cellubase/) is the first publicly accessible repository for BC genomics. CelluBase is designed to support comparative studies of cellulose-producing strains, in addition to providing the framework for future studies related to strain enhancement, cellulose yield improvement, and targeted metabolic engineering.

**Impact Statement**

CelluBase is the first of its kind resource for bacterial cellulose (BC) research, establishing a user-friendly platform for genomic and comparative analyses of bacteria from the genera *Komagataeibacter* and *Novacetimonas*. It has been designed to advance genomic studies of cellulose-producing bacteria, with the goal of expanding our understanding of BC biosynthesis and facilitating its use in biotechnological applications. The multi-scale analytical capabilities of this database – spanning individual gene characterization to genome-wide evolutionary analyses – make CelluBase a transformative tool for investigating the molecular mechanisms underlying BC biosynthesis. The platform is web-based, with all functions and tools integrated within the system, making genomic analyses accessible to users without prior expertise in complex -omics workflows.

One of the key features of CelluBase is a suite of tools designed for the characterization of cellulose synthase operon architecture and diversity. These tools allow users to interrogate genomic variation and identify conserved and divergent features within and between species. By linking genomic variation to functional potential, the platform may lead to the rational design of engineered strains, supporting the development of next-generation biomaterials. Beyond comparative analysis, CelluBase provides a foundation for synthetic biology applications by facilitating the identification of modular genetic elements, regulatory networks, and horizontally acquired traits that can be repurposed or redesigned. The comprehensive analytical framework and intuitive interface of CelluBase make it not only a versatile tool for academic research but also a valuable platform for industrial biotechnology, where it can be used to accelerate strain performance and biomaterial production.

## Data Availability

CelluBase database is available at https://cellubase.shinyapps.io/cellubase/.

## Introduction

Bacterial cellulose (BC) is a biopolymer synthesized by phylogenetically diverse bacterial taxa predominantly within the genus *Komagataeibacter* and the recently delineated *Novacetimonas* [1–3]. This extracellular polysaccharide, comprising β-1,4-linked glucopyranose residues, undergoes hierarchical self-assembly into highly hydrated, three-dimensional nanofibrous networks characterized by exceptional crystallinity, tensile strength, water retention capacity, and chemical purity [4–7]. In stark contrast to plant-derived cellulose, BC forms a purified cellulose biofilm, rather than being entrapped within the cell wall matrix [7]. The intrinsic physicochemical attributes of BC make it an exceptionally versatile material that has gained attention as an alternative biomaterial for biomedical applications, environmental engineering, acoustics, energy storage systems and sustainable textile manufacturing [7–10].

Cellulose biosynthesis in *Komagataeibacter* and *Novacetimonas* is governed by the highly conserved *bcsI* operon, which encodes the core enzymatic machinery required for glucan polymerization, crystallization and membrane extrusion [11–13]. The canonical genes *bcsA*, *bcsB*, *bcsC*, and *bcsD* execute specialized functions in cellulose polymerization, membrane anchorage, trans-envelope extrusion, and nascent fibre crystallization, respectively. Accessory genes, including *cmcAx*, *ccpAx,* and *bglAx*, provide additional regulatory complexity, modulating cellulose degradation, crystallization enhancement and glucan chain modification processes [12, 14, 15]. Genomic diversity within these taxa encompasses variable cellulose synthase operon architectures, with a subset of strains harbouring supplementary *bcsII* and *bcsIII* operons that govern modified cellulose synthesis, acylated cellulose production and surface adhesion properties critical for biofilm maturation.

Although the use of BC as a biomaterial has been on the rise, our understanding of the molecular mechanisms regulating cellulose biosynthesis, microfibril architecture, and performance limitations is still not well understood. Historically, conventional approaches to BC optimization have predominantly emphasized post-synthetic chemical modification or cultivation parameter manipulation – including the use of alternative carbon sources to enhance production yields [16–19]. Although moderate success has been achieved utilizing lab-based approaches for optimization, the integration of -omics data and genomic comparative tools to exploit cellulose-producing bacteria has largely remained unexplored [14, 20]. Unlike for other economically relevant groups of bacteria [e.g. the Pseudomonas Genome Database (https://pseudomonas.com/) and TubercuList (https://mycobrowser.epfl.ch/)], there are currently no dedicated tools or resources specific to cellulose-producing bacteria, limiting genomic analyses and comparative investigations of cellulose biosynthesis across diverse bacterial taxa.

To address the need for comprehensive genomic resources that support the investigation and genetic manipulation of cellulose-producing bacteria, we developed CelluBase – an integrated, user-friendly, application-driven database focused on the cellulose-producing genera *Komagataeibacter* and *Novacetimonas*. CelluBase is designed to be accessible to both academic and industry users, including those with limited experience in comparative -omics analyses. Within the CelluBase framework, all analyses are performed in an integrated environment enabling the rapid exploration of cellulose synthase gene organization, regulatory network architecture, metabolic pathway diversification and phylogenomic relationships across BC-producing lineages. Importantly, this resource provides a foundation for advances in synthetic biology and metabolic engineering by enabling the identification and rational redesign of genetic circuits and metabolic pathways underlying cellulose biosynthesis. We anticipate that CelluBase will facilitate the optimization of cellulose-producing bacteria by enabling comparative genomic analyses to identify candidate genes, biosynthetic pathways, and strain-specific traits associated with cellulose production. By linking genotype to functional output, CelluBase may support the rational design and engineering of microbial systems with enhanced or customizable cellulose production capabilities [21–25].

## Methods

CelluBase is a genomic repository of high-quality publicly available *Komagataeibacter* and *Novacetimonas* genome assemblies archived in NCBI databases (as of 5 October 2024). The genomes included in the database have passed stringent quality control (QC) parameters [1] and were analysed using standardized CheckM evaluation tools. Genomes with >95% completeness and <5% contamination were retained for the database. To address annotation inconsistencies inherent in diverse computational pipelines, genomes were re-annotated using Prokka v1.14.6 [26]. A systematic literature review was performed to determine the BC-producing capacity of the bacterium that passed sequencing QC.

The database was constructed using R with the Shiny web application framework for reactive interface development, incorporating shinydashboard and bslib packages for structured layout design and customizable theming protocols. The precomputed analytical outputs were stored hierarchically within module-specific directories corresponding to functional annotation categories. Data importation and preprocessing procedures utilize established R packages, including readr, readxl, dplyr and tidyr for standardized data manipulation workflows. Interactive tabular displays are rendered through the DT package, while statistical visualizations and graphical outputs are generated using ggplot2 [27], ggiraph, ggVennDiagram [28], circlize [29] and ComplexHeatmap [30] frameworks. Phylogenetic tree visualization employs ape and ggtree packages for evolutionary relationship display. Advanced genomic visualization capabilities integrate external JavaScript libraries: chromoMap.js [31] enables operonic structure representation, while CGView.js [32] provides comprehensive genome browser functionality. This hybrid computational architecture combining R-based analytical processing with JavaScript visualization libraries establishes seamless integration of data preprocessing, statistical analysis and interactive visualization within a unified user interface. CelluBase is an web-based platform optimized for genomic data exploration and comparative analysis workflows for *Komagataeibacter* and *Novacetimonas*.

## Results and discussion

### Database overview

**Fig. 1.**
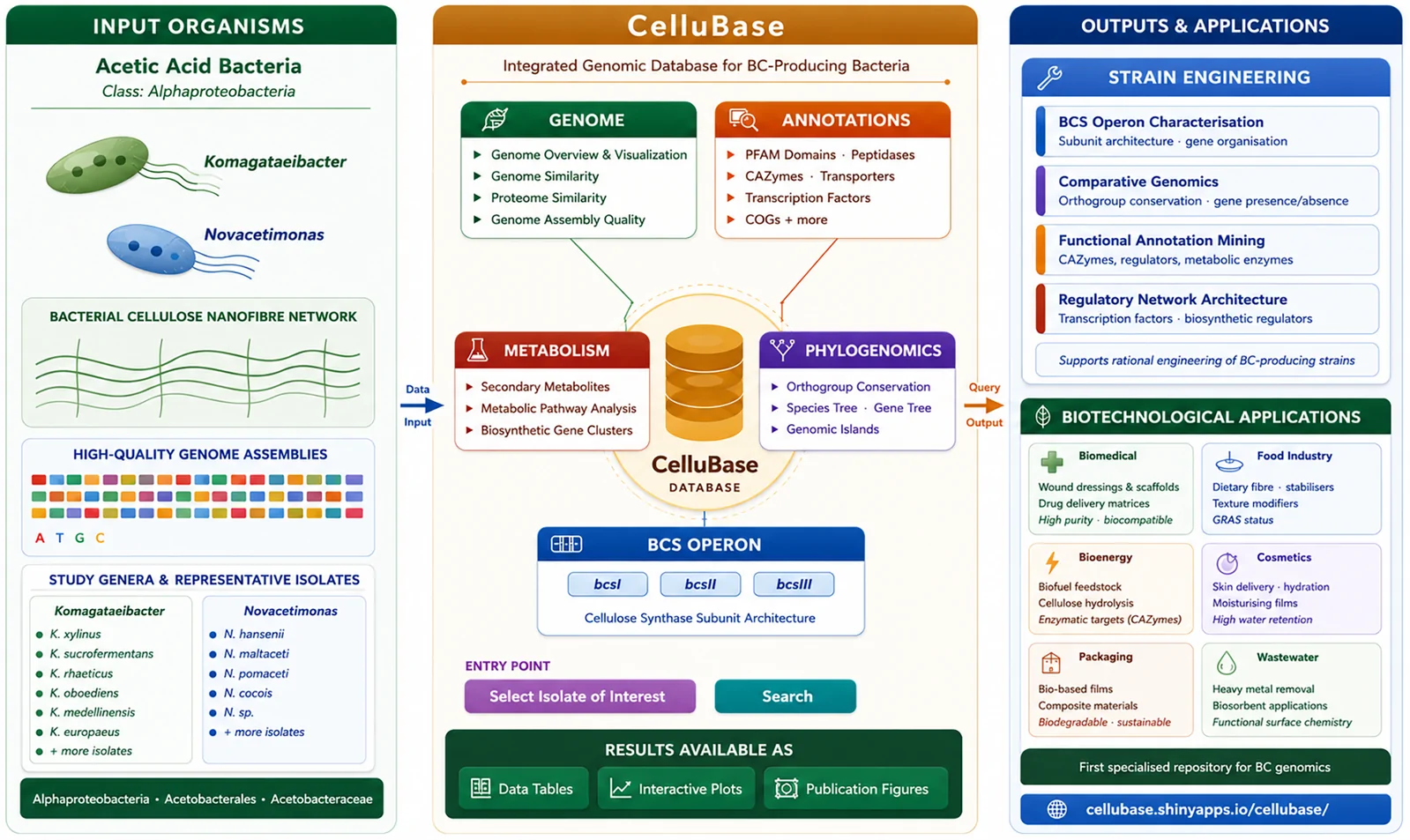
Schematic overview of the CelluBase database.

CelluBase is a curated, integrative database developed for the comparative and systematic investigation of cellulose-producing bacteria (Fig. 1). The database is designed to support both isolate exploration and comparative, multi-genome studies, with a focus on the diversity and organization of cellulose biosynthesis systems, functional annotation, and metabolic profiling. Users of CelluBase are first prompted to select an isolate of interest using the ‘Select Isolate’ function. For each isolate, comprehensive genome-level information is provided, including an interactive genome viewer that displays genome architecture and gene organization. Comparative genomic analysis tools to evaluate the relatedness and functional overlap across isolates are available in CelluBase (e.g. genome and proteome similarity). Protein-coding genes are annotated with Pfam domain assignments, enabling domain-centric interrogation of gene function. In addition, CelluBase provides users with key functional gene classes, including peptidases, carbohydrate-active enzymes (CAZymes), transporters, and transcription factors.

The metabolic capabilities of closely related cellulose-producing and non-cellulose-producing taxa can also be studied using CelluBase. For example, users can select a cellulose-producing strain and a closely related non-cellulose-producing strain to compare their annotated metabolic pathways, gene content, and bcs operons. These comparisons enable the identification of genes and pathways potentially associated with cellulose biosynthesis, while providing insights into functional diversification of closely related taxa.

Evolutionary relationships within and between *Komagataeibacter* and *Novacetimonas* can also be explored using the integrated phylogenomics function that links gene-level conservation with organismal history. Species-level phylogenies provide a taxonomic framework, while the identification of genomic islands highlights regions likely shaped by horizontal gene transfer. The conservation of orthogroups can be studied using multiple approaches, including pairwise comparisons, Venn diagrams, and gene tree reconstructions. Another central feature of CelluBase is the visualization tool for investigating cellulose biosynthesis operon structure. This feature annotates major cellulose synthase operon architectures to explore conservation and cellulose-producing potential, including the *bcsI*, *bcsII*, and *bcsIII* operons.

### Genome navigation and features

The Genome Overview interface, located under the Genome tab, provides a summary of all genome assemblies available in CelluBase. For each selected genome, a detailed report is provided that includes core identifiers (e.g. assembly and annotation accessions), species and strain designation, NCBI taxonomy identifiers and linked BioProject, BioSample and whole-genome sequencing (WGS) accessions. This information is important for cross-referencing public repositories and peer-reviewed publications. Genome quality and assembly characteristics are reported, including total sequence length, assembly level, chromosome and scaffold counts, contig and scaffold N50 values, sequencing platform, genome coverage, completeness and contamination estimates. Functional genomic summaries include total gene counts, protein-coding genes and pseudogenes. Metadata related to the isolation, cultivation and sequencing of each bacterium is provided when available. Importantly, CelluBase reports the cellulose-producing capacity for each bacterium based on available peer-reviewed literature.

**Fig. 2.**
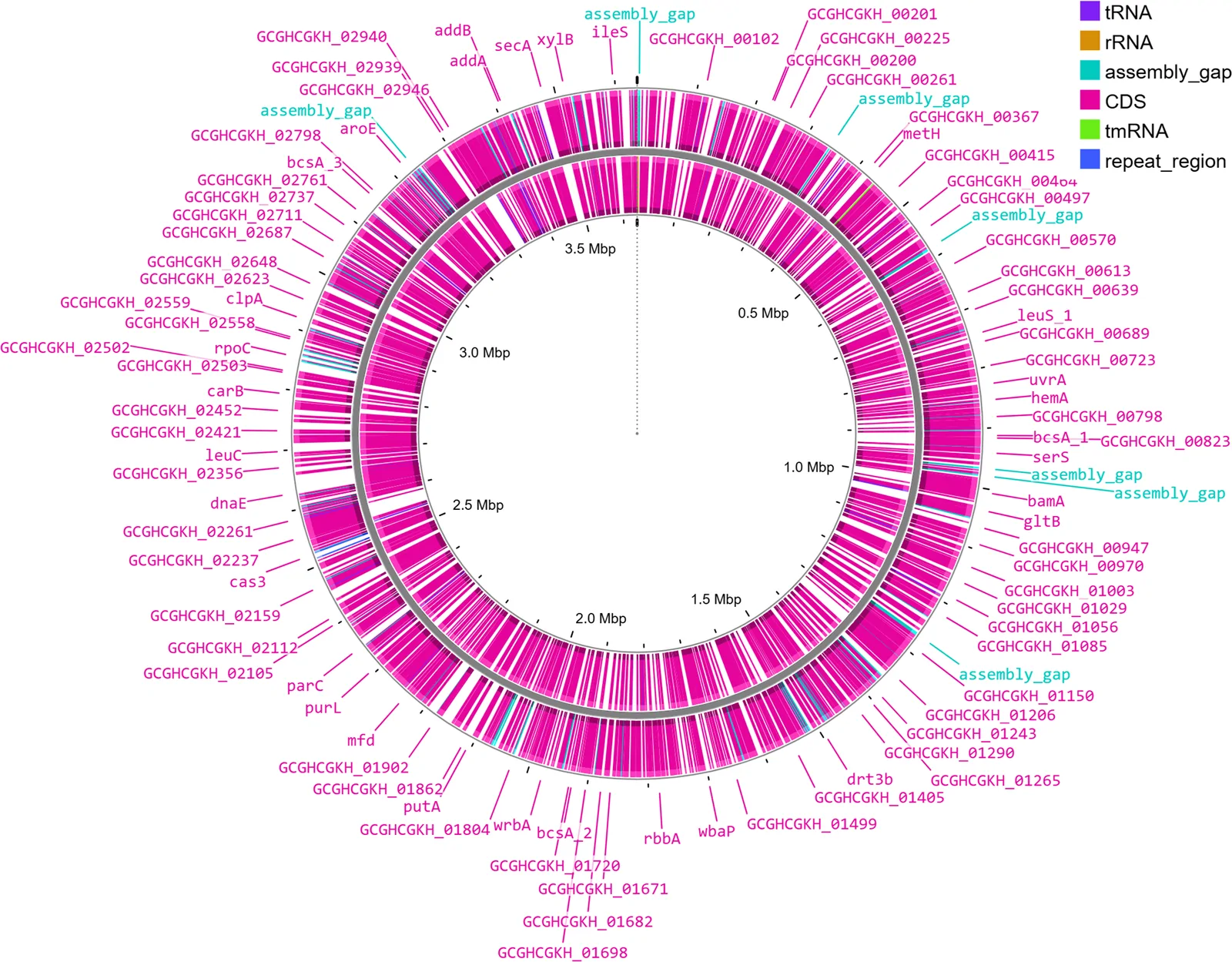
Circular genome visualization of *N. hansenii* ATCC 23769 generated using the Genome Visualization option. Annotated features include CDSs (pink), tRNA (purple), rRNA (brown), tmRNA (green), repeat regions (blue) and assembly gaps (cyan). Locus tags and gene names are displayed along the genome circumference. At the bottom of the Genome Visualization, a toolbar provides interactive functionalities, including zooming, panning, rotation, feature highlighting and download options, enabling detailed exploration and export of genome-wide information.

Beyond individual genome characterization, the Genome tab incorporates comparative analytical tools for Genome and Proteome Similarities (Fig. 2a, b). The Genome Similarity function provides average nucleotide identity (ANI) calculations – a standard metric for bacterial genome relatedness quantification. ANI computations utilize pyani [33] with MUMmer-based whole-genome alignment algorithms. Users select target genomes from the dropdown menu to generate pairwise similarity matrices visualized as interactive heatmaps with colour-coded identity values. Interactive functionality enables precise ANI value retrieval through cell-specific hover displays, while diagonal elements represent self-comparisons (identity=1.0). ANI thresholds ≥95% typically demarcate species-level taxonomic boundaries [1, 34], making this tool particularly valuable for taxonomic validation and phylogenomic analysis. For this function, there is the option to export the output as high-resolution, publication-ready figures.

The Proteome Similarity function complements genomic analyses by quantifying functional conservation through AAI calculations. AAI metrics assess protein-level conservation via alignments of shared single-copy orthologous gene sets. The platform implements FastAAI algorithms [35] optimized for large-scale proteome comparisons. The interactive heatmap visualizes pairwise AAI scores as colour-coded matrices reflecting protein conservation, which may be exported.

**Fig. 3.**
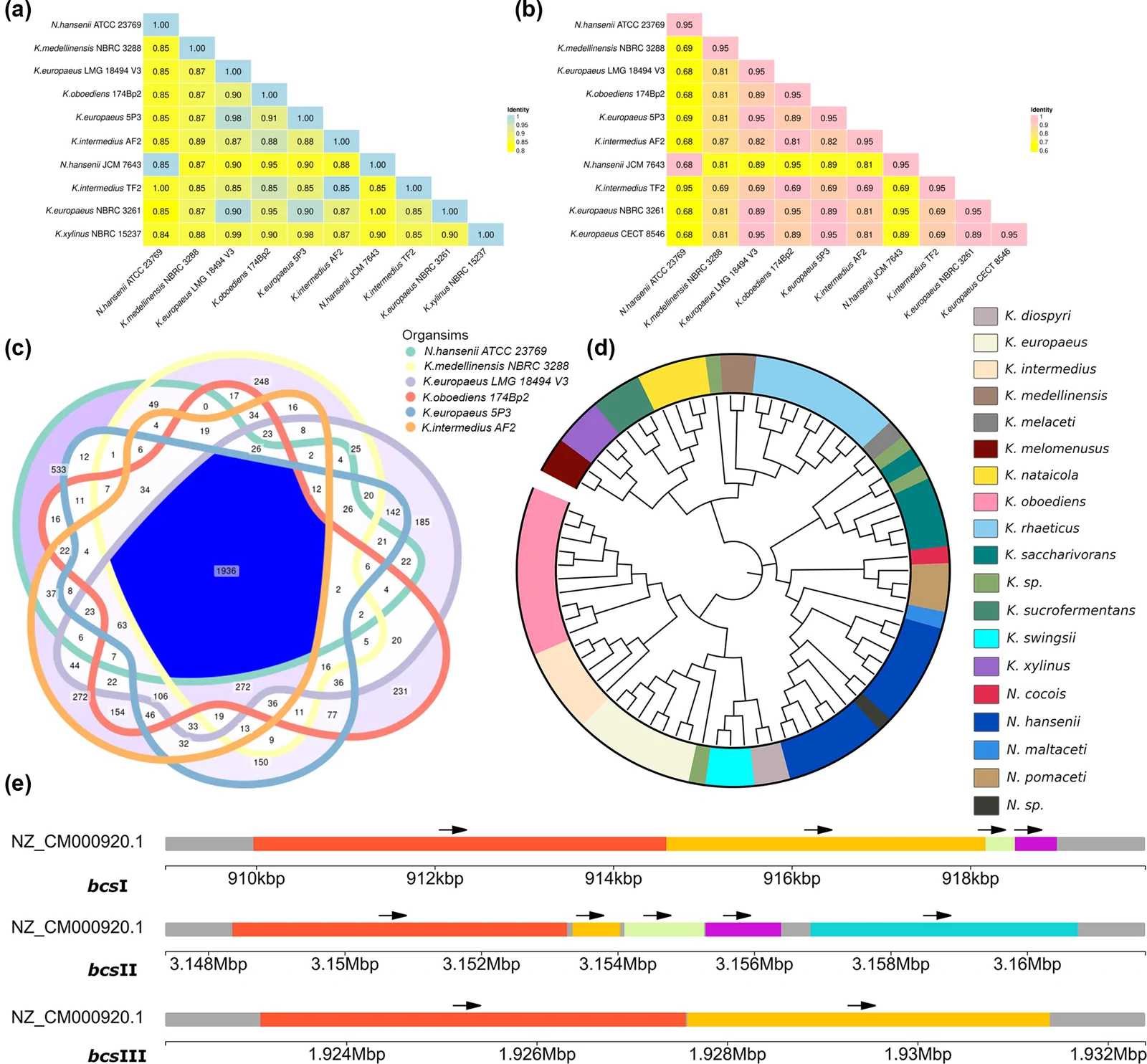
Comparative similarity analyses across the genomes available in CelluBase. (**a**) Pairwise ANI scores between selected genomes, visualized as a heatmap. (**b**) Average amino acid identity (AAI) scores across the same set of genomes, based on shared single-copy proteins using FastAAI. (**c**) Phylogenomics module of the database – Venn diagram view, showing shared and unique orthogroups across selected genomes. Users can select up to six genomes, generate an interactive Venn plot and export the results. (**d**) Phylogenetic relationships of species included in the database. The tree was inferred from concatenated multiple sequence alignments of single-copy orthologs (SCOs) and reconstructed using RAxML under a maximum-likelihood framework. (**e**) Interactive visualization of cellulose synthase operons in the database. Users can navigate between different operon types (*bcsI*–*bcsIII*) using the drop-down menu under the Cellulose Synthase Operon tab. Each operon is displayed with 1,000 bp flanking regions, showing gene organization, orientation and chromosomal coordinates. Hovering over a gene reveals its annotation (e.g. protein name and genomic location), while clicking on the gene redirects to the corresponding NCBI Protein entry for complete sequence and functional details. The example depicts the bcs operons from *Novacetimonas hansenii* ATCC 23769.

The Genome Visualization tool is used to generate interactive circular or linear chromosome mapping for genome architecture analysis (Fig. 3). Genomic features displayed include protein CDSs, tRNA genes, rRNA operons, transfer-messenger RNA sequences (tmRNAs), repetitive genomic elements and assembly discontinuities. Genomic coordinates, locus identifiers and gene nomenclature have been added around the chromosome periphery, facilitating feature identification. The output display is interactive, allowing for multi-scale zooming, directional panning across genomic regions, circular map rotation, view parameter reset options and selective feature display toggling. These features allow users to easily examine and compare genomes across multiple resolution scales, from chromosome-wide architectural overviews to fine-scale gene-level analysis.

### Multi-omics functional annotation framework

CelluBase enables users to explore protein domains, enzymatic activities, membrane transport systems, transcriptional regulatory networks, and orthologous gene clusters in both cellulose- and non-cellulose-producing bacteria from the genera *Komagataeibacter* and *Novacetimonas*. Users are also able to explore the molecular architecture underlying cellulose biosynthesis from multiple functional perspectives, facilitating both genome-wide surveys and detailed gene-level investigations. The annotation pipeline incorporates several bioinformatics tools and databases for functional predictions with the inclusion of external tools that support interpretability. For example, within the ‘Annotations’ tab, the Pfam Domains tool provides protein domain annotations generated using InterProScan [36]. For each predicted CDS within a selected organism, the interface displays associated Pfam and InterPro identifiers, domain descriptions and precise coordinate mappings within protein sequences. Hyperlinked database identifiers facilitate direct access to external repositories, including InterPro, Pfam and QuickGO, allowing the quick retrieval of protein family information, conserved domain architectures, functional annotations and associated Gene Ontology (GO) terms. Where available, GO annotations supply standardized predictions of molecular function, biological process and cellular component. This searchable and sortable interface supports efficient identification of functionally relevant protein domains and enables systematic exploration of protein features across complete proteomes.

The Peptidases classification tool provides systematic taxonomic organization of predicted proteolytic enzymes using the MEROPS database [37]. Protein sequences undergo BLASTp analysis applying stringent thresholds of 30% sequence identity, 50% query coverage and E-value cutoffs of 1×10⁻⁵ to ensure high-confidence predictions. The primary interface presents summary tables organizing peptidase families according to MEROPS clan designations, displaying clan codes, protein counts and catalytic mechanism descriptions encompassing serine or cysteine nucleophiles and specific active site architectures. Upon selecting a peptidase family of interest, detailed protein-level annotations, including database identifiers, gene nomenclature, Enzyme Commission (EC) numbers, MEROPS classifications and accession numbers, are provided – each hyperlinked to corresponding database entries for cross-referencing. GO annotations supplement functional interpretations, supporting downstream comparative and experimental analyses through this hierarchical navigation architecture.

The CAZyme annotation tool employs the dbCAN3 pipeline [38] for identification of carbohydrate metabolism potential. Users can select from three analytical approaches – HMMER, DIAMOND or dbCAN – for CAZyme family annotation through Hidden Markov Model profiling, sequence homology searches or an integrated strategy. The generated summary table classifies identified CAZyme families into functional groups, including glycoside hydrolases, glycosyltransferases, carbohydrate esterases, auxiliary activities, and carbohydrate-binding modules. Each entry displays family designations alongside associated gene counts. Selecting a count value directs users to a detailed results table listing gene identifiers, EC numbers and CAZyme classifications. The outputs are designed to help users link functional enzyme annotations with the corresponding genomic loci involved in polysaccharide biosynthesis, degradation, and modification.

The membrane transporter annotation tool can be used to predict transport proteins through homology searches against the Transporter Classification Database (TCDB) [39] using BLASTp. A default E-value threshold of 1×10⁻⁵ is applied to retain only high-confidence matches. The classification system organizes transport proteins according to TCDB’s major functional categories encompassing channels/pores, electrochemical potential-driven transporters, primary active transporters, group translocators, transmembrane electron carriers, accessory factors, and incompletely characterized systems. The summary table generated includes transporter gene counts per functional class, protein identifiers, genomic coordinates, sequence lengths, TCDB classifications, and substrate specificities where available.

The Transcription Factors tool utilizes TAPscan-classify [40] to classify transcriptional regulatory proteins based on conserved domain architectures aligned with established transcription factor families. The summary output table includes Pfam identifiers, associated protein counts and domain descriptions. Detailed protein-level views display identifiers, genomic coordinates, sequence characteristics and Pfam annotations, enabling the identification of regulatory hotspots and candidate genes for future functional studies within transcriptional regulatory networks.

The Clusters of Orthologous Groups (COGs) tool utilizes EggNOG-mapper [41] annotations for orthologous gene cluster analysis using bacterial-specific reference databases. Summary tables organize COG processes, categories, descriptions and CDS counts with percentage representations, featuring hyperlinked category labels to NCBI COG database entries for external validation. Gene-level resolution through count selection displays detailed annotations, including identifiers, functional descriptions, gene symbols, EC numbers, KEGG Orthology (KO) entries, Pfam domains, and Orthologous Group identifiers with external database hyperlinks, supporting both genome-wide functional profiling and detailed individual gene analysis.

### Biosynthetic gene cluster annotation and secondary metabolite prediction

CelluBase can be used for secondary metabolite biosynthetic pathway prediction using antiSMASH (version 8.0) [42]. This integrated feature allows users to explore genome-wide identification, annotation, and functional analysis of biosynthetic gene clusters (BGCs). CelluBase facilitates the detection of distinct BGCs, including non-ribosomal peptide synthetases (NRPS), polyketide synthases (PKS) and ribosomally synthesized and post-translationally modified peptides. Predicted gene clusters undergo systematic comparison against the Minimum Information about a Biosynthetic Gene cluster (MIBiG) reference database, which establishes standardized genomic specifications for experimentally validated BGCs.

The ‘Secondary Metabolites’ analytical tool is used to predict BGCs using the antiSMASH pipeline. For each genome represented in CelluBase, antiSMASH analyses are executed locally with resulting HTML-formatted summaries (index.html files) incorporated directly into the database framework. This design eliminates the need for users to switch between platforms, web browsers or to download supporting tools.

The interface provides a systematic overview of all detected BGCs categorized by biosynthetic class (NRPS, PKS, terpenes, etc.), accompanied by functional annotations and visualization of individual gene clusters. The output include genomic coordinate mapping, gene functional assignments, protein domain architectures, and comparative analyses with characterized clusters archived in established databases, including MIBiG [43]. A key feature is the interactive design of CelluBase, with users having the capability to engage with output files and zoom in/out of gene cluster diagrams and hyperlinked gene annotations.

CelluBase includes a metabolic pathway module for the exploration of the predicted metabolic repertoire of each genome. Functional annotations generated using EggNOG are mapped to KO identifiers and subsequently assigned to their corresponding metabolic pathways, allowing users to browse pathway-specific gene sets for individual genomes. To enhance biological relevance, CelluBase emphasizes bacterial metabolic and cellular pathways while excluding eukaryote-specific and disease-related pathways. This functionality facilitates comparative analyses of metabolic capabilities across cellulose-producing bacteria and provides a pathway-centric perspective of genome annotations.

### Phylogenomic analysis and evolutionary relationships

Understanding the evolutionary trajectories and genomic diversification patterns within cellulose-producing bacterial lineages requires sophisticated computational frameworks to resolve both phylogenetic relationships and recent adaptive events. Phylogenomic analyses integrate orthologous gene clustering, multiple sequence alignment algorithms, and robust phylogenetic reconstruction methods to elucidate genome content conservation, horizontal gene transfer events, and species-level evolutionary divergence [44]. These insights are directly relevant to metabolic engineering, strain enhancement and synthetic biology, as they enable the identification of target genes, pathways and adaptive traits that can be harnessed or redesigned to improve cellulose production and develop optimized microbial systems.

The Phylogenomics tool provides a comprehensive analytical suite for investigating evolutionary relationships across multiple hierarchical scales, encompassing orthogroup-level comparisons and species-level phylogenetic reconstructions. These integrated analyses combine gene clustering methodologies, multiple sequence alignment protocols, and phylogenetic inference algorithms to deliver in-depth insights into genome content conservation patterns and evolutionary divergence mechanisms across represented taxa.

The Orthogroup Conservation module establishes an interactive framework for comparative analysis of orthologous gene groups. Orthogroups, derived through OrthoFinder (version 2.5.4) [45] analyses, represent evolutionarily related gene sets originating from common ancestral sequences and provide fundamental units for investigating both conserved core functions and lineage-specific adaptations.

CelluBase also provides multiple options for data visualization, including Venn diagrams or Gene Trees. Venn diagrams accommodate up to six genome comparisons, displaying shared and unique orthogroup distributions between the selected assemblies (Fig. 2c). Each diagram allows users to very rapidly identify core, accessory, and strain-specific genes. Gene Tree showcases orthogroup evolutionary histories through phylogenetic reconstruction from multiple sequence alignments. The branching patterns reflect gene divergence, duplication events and species-specific variations, proving particularly valuable for investigating evolutionary trajectories of functionally critical genes. The Species Tree framework establishes global phylogenetic context through organismal-level evolutionary reconstruction (Fig. 2d). Species trees derive from concatenated multiple sequence alignments of single-copy orthologs using RAxML-NG [46] methodology, minimizing gene duplication and loss artefacts.

The Genomic Islands module enables rapid identification and exploration of DNA regions likely acquired through horizontal gene transfer using IslandPath-DIMOB algorithms [47], which detect atypical sequence composition and mobility-associated gene signatures. Results are presented in searchable, sortable tables detailing gene coordinates, strand orientation, protein identifiers and functional annotations. This streamlined framework supports both genome-wide surveys and targeted analysis of genes within these dynamic regions, while also providing valuable insights for synthetic biology, where horizontally acquired elements can inform the design and transfer of novel genetic functions and pathways.

### Cellulose synthase operons and biosynthetic architecture

BC biosynthesis is orchestrated by highly conserved yet structurally diverse operonic systems. These genes represent the core enzymatic clusters responsible for glucan polymerization, membrane translocation and extracellular fibre assembly. To elucidate the architectural variations of cellulose synthase operons, a Cellulose Synthase Operon comparison tool is available. This tool implements a specialized analytical framework, building upon established methodologies [1], for systematic characterization of gene clusters governing BC biosynthesis across represented genomes. The operons – designated *bcsI* (*bcsABCD*), *bcsII* (*bcsAB*₂*XYC*₂) and *bcsIII* (*bcsAB*₃*C*₃) – are displayed as graphical representations of the gene clusters and flanking genomic regions (1,000 bp extensions), allowing users to quickly assess chromosomal organization and potential regulatory elements (Fig. 2e). Colour-coded blocks are used to represent gene orientation, boundaries and chromosomal positioning. Additional gene-specific information (including gene nomenclature, protein designations, and precise genomic coordinates) is available by hovering the pointer over the region of interest. Navigation between distinct operon types can be achieved using the built-in dropdown menu.

### Future directions

Future development of CelluBase will focus on expanding its comparative genomics, functional annotation, and multi-omics capabilities. Planned enhancements include comparative visualization of pathway-associated gene presence and absence across multiple genomes, enabling more comprehensive analyses of cellulose biosynthesis, carbohydrate metabolism, and other bacterial metabolic processes. Support for user-submitted genome analyses will allow newly identified cellulose-producing bacteria to be compared directly with existing datasets, facilitating the discovery and characterization of novel cellulose-producing strains. Future releases will also incorporate historical taxonomic names and nomenclature changes to improve links with legacy literature and genome records.

Expansion of the database to include additional cellulose-producing genera and emerging taxa from environmental surveys will broaden the evolutionary landscape represented in CelluBase and facilitate the discovery of previously uncharacterized cellulose biosynthetic variants. The integration of pangenomic analyses, together with transcriptomic and metabolomic datasets, will establish a comprehensive multi-omics framework for investigating the genetic regulation, metabolic networks, and environmental responses underlying BC production. In parallel, machine learning approaches trained on experimentally validated phenotypic data will be explored to predict cellulose production potential and identify promising strains for biotechnological applications. Finally, allowing researchers to contribute new genomic data, together with standardized procedures for data curation and quality control, will support the continued expansion and utility of CelluBase as a resource for studying cellulose-producing bacteria.

## Abbreviations

AAI: average amino acid identity
ANI: average nucleotide identity
BC: bacterial cellulose
BGCs: biosynthetic gene clusters
CAZymes: carbohydrate-active enzymes
COGs: Clusters of Orthologous Groups
EC: Enzyme Commission
GO: Gene Ontology
KO: KEGG Orthology
MIBiG: Minimum Information about a Biosynthetic Gene cluster
NRPS: non-ribosomal peptide synthetases
PKS: polyketide synthases
QC: quality control
SCOs: single-copy orthologs
TCDB: Transporter Classification Database
tmRNAs: transfer-messenger RNA sequences
WGS: whole-genome sequencing

## References

[1] Akhoon BA, Qiao Q, Stewart A, Chen J, Rodriguez Lopez CM (2025). Pangenomic analysis of the bacterial cellulose-producing genera Komagataeibacter and Novacetimonas. Int J Biol Macromol.

[2] Brandão PR, Crespo MTB, Nascimento FX (2022). Phylogenomic and comparative analyses support the reclassification of several *Komagataeibacter* species as novel members of the *Novacetimonas gen. nov*. and bring new insights into the evolution of cellulose synthase genes. Int J Syst Evol Microbiol.

[3] Gao M, Li J, Bao Z, Hu M, Nian R (2019). A natural in situ fabrication method of functional bacterial cellulose using a microorganism. Nat Commun.

[4] Cheng KC, Catchmark JM, Demirci A (2009). Effect of different additives on bacterial cellulose production by *Acetobacter xylinum* and analysis of material property. Cellulose.

[5] Girard VD, Chaussé J, Vermette P (2024). Bacterial cellulose: a comprehensive review. J Appl Polym Sci.

[6] Seddiqi H, Oliaei E, Honarkar H, Jin J, Geonzon LC (2021). Cellulose and its derivatives: towards biomedical applications. Cellulose.

[7] Wang J, Tavakoli J, Tang Y (2019). Bacterial cellulose production, properties and applications with different culture methods – a review. Carbohydrate Polymers.

[8] Jozala AF, de Lencastre-Novaes LC, Lopes AM, de Carvalho Santos-Ebinuma V, Mazzola PG (2016). Bacterial nanocellulose production and application: a 10-year overview. Appl Microbiol Biotechnol.

[9] Muiruri JK, Yeo JCC, Zhu Q, Ye E, Loh XJ (2023). Bacterial cellulose: recent advances in biosynthesis, functionalization strategies and emerging applications. European Polymer Journal.

[10] Rosson L, Tan B, Best W, Byrne N (2024). Applications of regenerated bacterial cellulose: a review. Cellulose.

[11] Bimmer M, Mientus M, Klingl A, Ehrenreich A, Liebl W (2022). The roles of the various cellulose biosynthesis operons in *Komagataeibacter hansenii* ATCC 23769. Appl Environ Microbiol.

[12] Bimmer M, Reimer M, Klingl A, Ludwig C, Zollfrank C (2023). Analysis of cellulose synthesis in a high-producing acetic acid bacterium *Komagataeibacter hansenii*. Appl Microbiol Biotechnol.

[13] Römling U, Galperin MY (2015). Bacterial cellulose biosynthesis: diversity of operons, subunits, products, and functions. Trends Microbiol.

[14] Florea M, Hagemann H, Santosa G, Abbott J, Micklem CN (2016). Engineering control of bacterial cellulose production using a genetic toolkit and a new cellulose-producing strain. Proc Natl Acad Sci U S A.

[15] Sumini M, Andrade GJS, Tischer CA, Kobayashi RKT, Nakazato G (2025). Production of bacterial cellulose by Komagataeibacter xylinus: biochemistry, synthesis and applications. Cellulose.

[16] Akhoon BA, Corley J, Tan JW, Magnani R, Schendel RR (2026). Carbon source-induced changes in the transcriptional landscape of Novacetimonas hansenii. Bioresour Technol.

[17] El-Gendi H, Taha TH, Ray JB, Saleh AK (2022). Recent advances in bacterial cellulose: a low-cost effective production media, optimization strategies and applications. Cellulose.

[18] Mohammadkazemi F, Azin M, Ashori A (2015). Production of bacterial cellulose using different carbon sources and culture media. Carbohydr Polym.

[19] Tureck BC, Hackbarth HG, Neves EZ, Garcia MCF, Apati GP (2021). Obtaining and characterization of bacterial cellulose synthesized by *Komagataeibacter hansenii* from alternative sources of nitrogen and carbon. Matéria (Rio J).

[20] Moradi M, Jacek P, Farhangfar A, Guimarães JT, Forough M (2021). The role of genetic manipulation and in situ modifications on production of bacterial nanocellulose: a review. Int J Biol Macromol.

[21] Caro-Astorga J, Walker KT, Herrera N, Lee KY, Ellis T (2021). Bacterial cellulose spheroids as building blocks for 3D and patterned living materials and for regeneration. Nat Commun.

[22] Laurent JM, Jain A, Kan A, Steinacher M, Enrriquez Casimiro N (2024). Directed evolution of material-producing microorganisms. Proc Natl Acad Sci USA.

[23] Núñez D, Oyarzún P, González S, Martínez I (2024). Toward biomanufacturing of next-generation bacterial nanocellulose (BNC)-based materials with tailored properties: a review on genetic engineering approaches. Biotechnol Adv.

[24] Shin S, Kwak H, Shin D, Hyun J (2019). Solid matrix-assisted printing for three-dimensional structuring of a viscoelastic medium surface. Nat Commun.

[25] Walker KT, Li IS, Keane J, Goosens VJ, Song W (2025). Self-pigmenting textiles grown from cellulose-producing bacteria with engineered tyrosinase expression. Nat Biotechnol.

[26] Seemann T (2014). Prokka: rapid prokaryotic genome annotation. Bioinformatics.

[27] Wickham H (2011). Wiley interdiscip. Rev Comput Stat.

[28] Gao CH, Yu G, Cai P (2021). ggVennDiagram: an intuitive, easy-to-use, and highly customizable R package to generate venn diagram. *Front Genet*.

[29] Gu Z, Gu L, Eils R, Schlesner M, Brors B (2014). Circlize implements and enhances circular visualization in R. Bioinformatics.

[30] Gu Z (2022). Complex heatmap visualization. Imeta.

[31] Anand L, Rodriguez Lopez CM (2022). ChromoMap: an R package for interactive visualization of multi-omics data and annotation of chromosomes. *BMC Bioinformatics*.

[32] Stothard P, Grant JR, Van Domselaar G (2019). Visualizing and comparing circular genomes using the CGView family of tools. Briefings in Bioinformatics.

[33] Pritchard L, Glover RH, Humphris S, Elphinstone JG, Toth IK (2016). Genomics and taxonomy in diagnostics for food security: soft-rotting enterobacterial plant pathogens. Anal Methods.

[34] Thompson CC, Chimetto L, Edwards RA, Swings J, Stackebrandt E (2013). Microbial genomic taxonomy. BMC Genom.

[35] Gerhardt K, Ruiz-Perez CA, Rodriguez-R LM, Jain C, Tiedje JM (2025). FastAAI: efficient estimation of genome average amino acid identity and phylum-level relationships using tetramers of universal proteins. Nucleic Acids Res.

[36] Blum M, Chang H-Y, Chuguransky S, Grego T, Kandasaamy S (2021). The InterPro protein families and domains database: 20 years on. Nucleic Acids Res.

[37] Rawlings ND, Barrett AJ, Finn R (2016). Twenty years of the MEROPS database of proteolytic enzymes, their substrates and inhibitors. Nucleic Acids Res.

[38] Zheng J, Ge Q, Yan Y, Zhang X, Huang L (2023). dbCAN3: automated carbohydrate-active enzyme and substrate annotation. Nucleic Acids Res.

[39] Saier MH, Reddy VS, Moreno-Hagelsieb G, Hendargo KJ, Zhang Y (2021). The Transporter Classification Database (TCDB): 2021 update. Nucleic Acids Res.

[40] Petroll R, Varshney D, Hiltemann S, Finke H, Schreiber M (2025). Enhanced sensitivity of TAPscan v4 enables comprehensive analysis of streptophyte transcription factor evolution. Plant J.

[41] Cantalapiedra CP, Hernández-Plaza A, Letunic I, Bork P, Huerta-Cepas J (2021). eggNOG-mapper v2: functional annotation, orthology assignments, and domain prediction at the metagenomic scale. Mol Biol Evol.

[42] Blin K, Shaw S, Vader L, Szenei J, Reitz ZL (2025). antiSMASH 8.0: extended gene cluster detection capabilities and analyses of chemistry, enzymology, and regulation. Nucleic Acids Res.

[43] Terlouw BR, Blin K, Navarro-Muñoz JC, Avalon NE, Chevrette MG (2023). MIBiG 3.0: a community-driven effort to annotate experimentally validated biosynthetic gene clusters. Nucleic Acids Res.

[44] Akhoon BA, Gupta SK, Dhar MK (2023). Dissecting the genome, secretome, and effectome repertoires of Monilinia spp.: the causal agent of brown rot disease: a comparative analysis. Postharvest Biol Technol.

[45] Emms DM, Kelly S (2019). OrthoFinder: phylogenetic orthology inference for comparative genomics. Genome Biol.

[46] Kozlov AM, Darriba D, Flouri T, Morel B, Stamatakis A (2019). RAxML-NG: a fast, scalable and user-friendly tool for maximum likelihood phylogenetic inference. Bioinformatics.

[47] Bertelli C, Brinkman FSL (2018). Improved genomic island predictions with IslandPath-DIMOB. Bioinformatics.

